# Development of catalyst complexes for upgrading biomass into ester-based biolubricants for automotive applications: a review

**DOI:** 10.1039/c7ra11824d

**Published:** 2018-02-01

**Authors:** Md. Anwar Hossain, Mohammad Anwar Mohamed Iqbal, Nurhidayatullaili Muhd Julkapli, Pei San Kong, Juan Joon Ching, Hwei Voon Lee

**Affiliations:** Nanotechnology & Catalysis Research Centre (NANOCAT), Institute of Postgraduate Studies, Universiti Malaya 50603 Kuala Lumpur Malaysia leehweivoon@um.edu.my +603-7957-6956 +603-7967-6954; Department of Chemistry, Rajshahi University of Engineering & Technology Rajshahi 6204 Bangladesh; School of Chemical Sciences, Universiti Sains Malaysia 11800 Penang Malaysia; Department of Chemical Engineering, Faculty of Engineering, Universiti Malaya 50603 Kuala Lumpur Malaysia; Laboratoire de Génie Chimique (Labège), BP84234 Campus INP-ENSIACET 4 allée Emile Monso 31432 Toulouse Cedex 4 France

## Abstract

Biomass-derived oils are recognised as the most promising renewable resources for the production of ester-based biolubricants due to their biodegradable, non-toxic and metal adhering properties. Homogeneous acid catalysts have been conventionally used in catalytic esterification and transesterification for the synthesis of ester-based biolubricants. Although homogeneous acid catalysts encounter difficulty during phase separation, they exhibit superior selectivity and good stereochemistry and regiochemistry control in the reaction. Consequently, transition metal complex catalysts (also known as homogeneous organometallic catalysts) are proposed for biolubricant synthesis in order to achieve a higher selectivity and conversion. Herein, the potential of both homogeneous transition metal complexes and heterogeneous supported metal complexes towards the synthesis of biolubricants, particularly, in esterification and transesterification, as well as the upgrading process, including hydrogenation and *in situ* hydrogenation–esterification, is critically reviewed.

## Abstract 2

The graphical abstract reflects the content of the entire paper; catalyst complexes for upgrading biomass to ester-based biolubricants.

## Graphical abstract

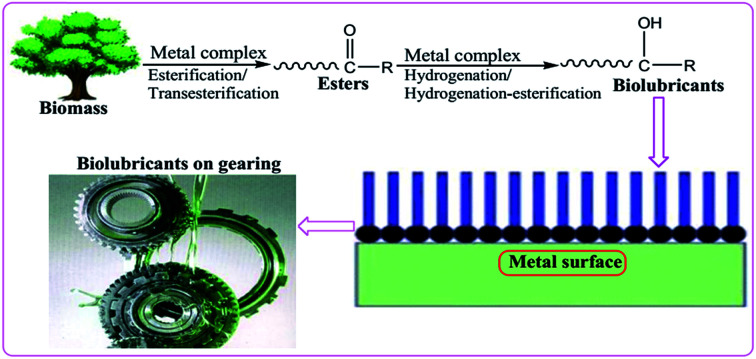

## 1 Introduction

Automotive-type lubricants are utilised to reduce friction and to moderate the heat produced when two surfaces are in mutual contact.^1^ The lubricants can form a thin film between the two sliding parts, which generates a slippery surface for the metal parts to move easily.^2^ Generally, lubrication can prolong the wear and tear duration of machinery and significantly reduce the surface deformation of the parts. Besides acting as a coolant for the sliding surfaces, lubricants also act as a transport medium for foreign particles and forces on the parts of machinery.

Generally, petroleum-based oils or mineral oils have been used as lubricants in automobiles and machinery for several decades. The continuous depletion of mineral oils has raised the attention of scientists towards the utilisation of renewable biomass-based lubricants.^3^ It has been widely reported that the petroleum-based oils can lead to serious groundwater pollution,^4^ soil pollution, surface water contamination, air pollution, and agricultural material and food contamination.^5^ Moreover, gaseous CO, CO_2_, NO_*x*_ and SO_*x*_ are liberated together with nanoparticle traced metals (Hg, Ca, P, Zn, Mg, and Fe) during the combustion of mineral oil, which results in a negative impact on the environment.^6^ It is reported that mineral oil is carcinogenic as continuous inhalation of this lubricant emission may cause inflammatory and analgesic effects on the human respiratory system.^7,8^ In addition, by-products derived from the degradation of the lubricants are toxic and can lead to soil infertility.

Ester-based biolubricants are greener, renewable, non-toxic and emit zero greenhouse gas.^9^ They can be used as additives for anti-oxidants, viscosity index promoters, pour point mitigation, detergents and emulsion stabilisers. For industrial application, these biolubricants are suitable to be applied for complete fluid lubrication, boundary lubrication, and extreme pressure lubrication.

Notably, bioester-based lubricants are conventionally synthesised by reacting long-chain fatty acids (derived from hydrolysis of plant oil) with polyols (*e.g.* trimethylolpropane, neopentyl glycol, pentaerythritol) in the presence of liquid acid or Lewis acid catalysts by esterification. In contrast, the reaction between triglyceride-based feedstock (plant oil or animal fat) and alcohols/polyols occurs *via* the transesterification process in the presence of acid or base catalyst.^10–12^ Recently, a new technology has been developed to upgrade the performance of ester-based biolubricants (especially in oxidation stability) *via* hydrogenation, and/or *in situ* hydrogenation–esterification in which molecular hydrogen is used at a high temperature and pressure in the presence of homogeneous or heterogeneous catalysts. These types of reactions aim to reduce the unsaturated bonding presence in the bioester into a saturated product for better stability of the product under extreme conditions and long-term storage.

Recent development focused on the production of various characteristics of biolubricant products has provided potential substitutes to mineral-based lubricants for automotive applications.^13,14^ Nevertheless, to the best of our knowledge, there is a lack of comprehensive study on the usage of homogeneous transition complex catalysts in biolubricant synthesis.^15,16^ As derived from the literature, a homogeneous-type transition metal complex catalyst is a potential catalyst for ester production, mainly due to its simple preparation step, large surface area, good molecular dispersion between reactants, and sufficient pore volume.^17^ In contrast, the heterogeneous-type metal complex catalyst is scarce in the catalytic synthesis of biolubricants when long-chain/polyhydric alcohols are employed in the reaction due to the occurrence of undesirable side products, low selectivity and soap formation in the process.^18^ Consequently, the catalytic activity of homogeneous transition metal complexes and the supported metal complexes (heterogeneous) towards the production of biolubricants is critically reviewed in this study. In addition, this review outlines the future prospects of and global demand for biolubricants.

### 1.1 Global demand for lubricant usage

**Fig. 1:**
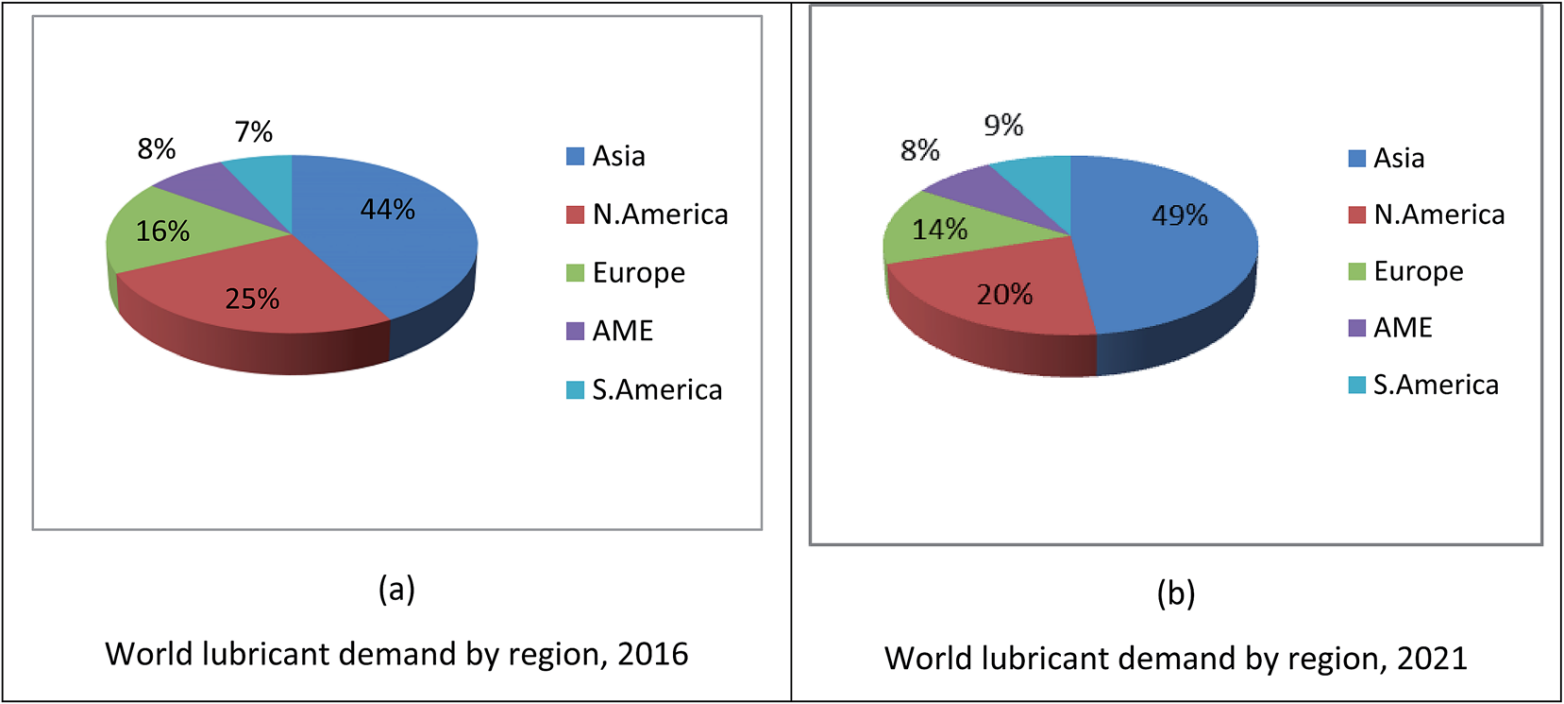
World lubricant demand by region for 2016 and 2021.

The global demand for lubricants is expected to increase 2.0% per year to 45.40 million metric tons by 2020.^6^ This growth is projected to increase as the demand for engine oils for new motor vehicles increases. The fastest demand is projected for the Asia and Pacific regions, where number of motor vehicles and industrialization are increasing. The demand for lubricants in Central and South America and the Middle East/Africa region is also expected to increase due to the increasing number of manufacturing units, rising motor vehicle manufacturing and proprietary rates. Fig. 1 shows the world demand of lubricant usage in 2016 (a) and world lubricant demand forecast for the year 2021 (b).^19,20^ According to a published report by Kline & Company, the collected data in 2016 indicated that the demand for lubricants is increasing in Asia and South America (S. America), whereas, it is decreasing in North America (N. America) and Europe and is constant in Africa/Middle East (AME). The reasons are the strong economic growth and increase in vehicle ownership rate and industrialisation, which result in an increase in demand for lubricants. Besides, another analysis reported by RPS Energy (a multinational energy and environmental consultancy company) stated that the demand of lubricants is increasing continuously in the Asian region due to economic prospects, vehicle ownership and large-scale GDP growth. As GDP growth per capita income is rising in Asia and Middle East/Africa regions compared to that in North America and Europe, there is an increase in vehicle ownership rate, thus leading to an increase in lubricant demand (Table 1).^19,20^

**Table 1:** Regional economic prospects and vehicle ownership for lubricant growth (2012–2023) [source: RPS, EIU, World Bank – estimates range from low to high with a global average of (0.1%) to 1.5%]

Asia Pacific		Middle East & Africa		North America		Europe	
GDP growth (avg)	4.0%	GDP growth (avg)	4.0%	GDP growth (avg)	2.4%	GDP growth (avg)	1.7%
Per capita (avg)	3.6%	Per capita (avg)	2.3%	Per capita (avg)	1.6%	Per capita (avg)	1.5%
Lubes growth	1–2%	Lubes growth	0.5–2%	Lubes growth	(1%)–1%	Lubes growth	(1%)–0.5%

### 1.2 Type of automotive lubricants

Besides providing a slippery surface for the moving parts, a good automotive lubricant must be able to remain in the liquid state within a wide range of temperatures. For instance, the lubricant oil should have a high boiling temperature with low freezing points, so that it can stay on the metallic surface in the liquid form for long periods. Indeed, the viscosity index of lubricant oils is the most important property to determine it's application in automotive usage such as resistant to flow, adherence on the surfaces, and changes in temperature and pressure. Thermal and oxidation stability indicates the stability of the lubricating oils at higher temperatures and pressures. Tables 2 and 3 show the categories for different types of automotive lubricants that are measured by various factors.

**Table 2:** Categories of lubricant base oil by API standards^21–25^

Sources	Category of base oils	Sulphur (%)	Saturation (%)	Viscosity index
**Categories of base oils, API**				
Mineral	Group-I base oils are prepared through solvent extraction, hydro-finishing and catalytic dewaxing processes at temperature ranges of 305–423 °C	Greater than 0.03	Less than 90	80–120
	Group-II (hydrotreated) base oils are highly stable and have better anti-oxidation properties, since all the carbon molecules are highly saturated	Less than 0.03	Greater than 90	80–120
	Group-III (hydrocracked) base oils are synthesized by special processes called isohydromerization and are severely hydrocracked at elevated temperature and pressure	Less than 0.03	Greater than 90	Greater than 120
Synthetic^a^	Group-IV base oils are prepared by a reaction called manufacturing or synthesizing. These oils can be used in a wide range of temperatures such as in crucial cold conditions, extreme heat applications and also suitable under extreme pressure	PAO synthetic lubricants		
	Group-V base oils including silicone, polyolester, phosphate ester, biolubes and polyalkylene glycol. Currently, these oils are mixed with other base stocks for improving the oil's performance	All other base oils rather than groups I, II, III, or IV		

a Synthetic oils have high a viscosity index (130–210) and high saturation (>92%) and contain a smaller amount of sulphur (0.003 wt%).^26^

**Table 3:** Categorization of lubricants depending on various properties^27–30^

**Physical appearance**	
Solid	Lubricants form a thin film of material on the metal surface. They comprise organic or inorganic compounds, for example, graphite, molybdenum disulphide and cadmium disulphide
Semi-solid	The liquid is dissolved in solid and sometimes additives are added, for example, grease
Liquid	The examples of liquid oils are as follows: vegetable oils, petroleum oils, and synthetic and animal oils

**Resources of base oil**	
Natural sources	These base oils are obtained from vegetable oils and/or animal fats and are called natural oils
Refined sources	Oils obtained from crude petroleum sources; examples are paraffinic oils and aromatic and naphthenic oils
Synthetic oils sources	They are highly synthesized as final reaction products, such as synthetic esters, silicones, and polyalphaolefins

**On the basis of application**	
Automotive oils/fluids	These oils are commonly used for automobile application and transportation sectors, *e.g.*, engine oils, gear-box oils, transmission fluids, and hydraulic brake fluids
Industrial instrument oils	They are used for industrial goals such as compressor fluids, machine oils, hydraulic fluids, and metal-working oils
Special fluids	These fluids are used in special cases according to definite applications such as white fluids, process fluids, and instrumental fluids

## 2 Biolubricants

Biolubricants are generally obtained from biomass or naturally occurring sources like soybean, palm, sunflower, coconut, and rapeseed oils.^16^ A biolubricant is an ester of glycerides, which is still limited for application as a lubricant as their freezing point is quite high, and they are easily oxidised. Biolubricants can be prepared using long-chain alcohols/polyols with fatty acids that fulfil the toxicity and biodegradability criteria (refer to Table 4). Typically, biolubricants are biodegradable and release less carbon and greenhouse gases compared to petroleum-based lubricants. As a result, biolubricants evaporate slowly compared to petroleum oil and adhere to metal surfaces strongly even under extreme conditions for a long time. A biolubricant's adhering properties on metal surfaces and performance can also be increased by additives and can be used under abnormal conditions. For instance, fatty acid such as oleic acid, linoleic acid, butanoic acid, levulinic acid and palmitic acid react with long-chain/polyhydric alcohols forming mono-, di-, triester-based biolubricants. In general, conventional biolubricant oils found in the market are derived from biomass, such as castor oil, olive oil, coconut oil, sperm oil, rapeseed oil, rosin oil, palm oil, neatsfoot oil, and seal and whale oils. Commonly, mineral oil is blended with biolubricant base oil to increase the lube performance.

**Table 4:** Different physical properties of synthetic biolubricants^33–36^

Polyol	Fatty acids	Viscosity index	Pour point (°C)	Flash point (°C)	Oxidation stability	Biodegradability (%)
NPG	Oleic acid	207	−24	272	175	98
	Acetic acid	135	−22	275	181	97
TMP	Oleic acid	190	−39	289	189	95
	Levulinic acid	150	−25	280	184	99
	Caprylic acid	114	−45	285	178	94
PE	Oleic acid	141	−21	>300	177	98
Gly	Oleic acid	180	−28	278	176	96
	Butanoic acid	160	−25	275	179	98

### 2.1 Synthesis of biolubricants

#### (I) Esterification

Generally, ester-based biolubricants can be prepared *via* esterification reactions between polyhydric alcohols with fatty acids in the presence of acid catalyst. The employed polyols such as neopentyl glycol (NPG), trimethylolpropane (TMP), pentaerythritol (PE) and glycerol (Gly) are able to render different characteristics to the biolubricant product (*e.g.* high viscosity, low freezing point, low pour point and high flash point). Meanwhile, a different carbon chain number of carboxylic acids, such as acetic acid, propionic acid, oleic acid, valeric acid, caprylic acid and levulinic acid, can be used for the esterification process. Generally, the length of the fatty acid chain for biolubricant synthesis are in the range of C_12_–C_24_. The two major factors of fatty acids that affect the properties of biolubricants are the carbon chain length and number of double bonds in the fatty chain.^31^ A longer chain length of fatty acids produces biolubricants with a higher melting point and viscosity, whereas higher double bonds alter the property of biolubricants by lowering their melting points and viscosity and decreasing their oxidative stability.^1^ Thus, different fatty acids and polyol feedstocks render different characteristics to biolubricant products for automotive and industrial usage.^32^
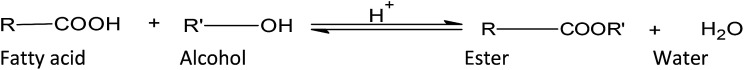


#### (II) Transesterification

Transesterification reaction is another conventional process for synthesising ester-based biolubricants using triglyceride-based feedstock (plant-based or animal-based oil) and alcohols in the presence of catalyst. For the transesterification process, different types of triglyceride-based oils including soybean, palm, andiroba, piqui and cumaru oils are involved in the methanolysis in the presence of different Lewis acid catalysts. In this review, we tried to describe the Lewis acidity of catalysts, which influences the transesterification reaction. For this reason, we included some metal salts, especially Sn(ii) and Sn(iv) based complexes having Lewis acidity, with different ligands, which have been critically reviewed. Their catalytic activity was also compared with that of conventional mineral acid H_2_SO_4_ catalyst. The main advantage of the Sn based-complex catalyst is that it is highly soluble in the oil phase and gives the maximum yield, but sometimes catalyst separation is difficult. The general transesterification reaction in the presence of catalyst is as follows:
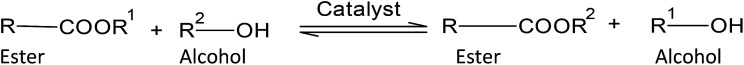


#### (III) Hydrogenation

Hydrogenation of esters is an important upgrading process for the synthesis of biolubricants, surfactants, plasticisers, and fatty alcohols, which have broad applications in agrochemicals, pharmaceuticals, and fine chemicals. The reduction of esters and ketones is by employing H_2_ at higher temperature and pressure for producing ester-based biolubricants that have a significant importance in our daily life. The normal ester-based lubricants have a high oxygen content and unsaturation level, which are not suitable for engine oils or fine chemicals, and are not biodegradable. On the other hand, ester-based lubricants after hydrogenation process, increase the hydrogen content and saturated carbon chain can be used as engine oils, which might satisfy all types of lubrication. The high-potential catalysts for the reduction process of the carbonyl group are present in the ester including the precious Ru, Rh, Os, Pt, and Au metal-based complexes. However, the high price and limited availability of the valuable metals lead to the exploration of the highly abundant and less expensive active metal complexes, *e.g.*, transition metal (Cu, Ni, Co and Fe) based complexes. The general ester hydrogenation in the presence of the catalyst is as follows:
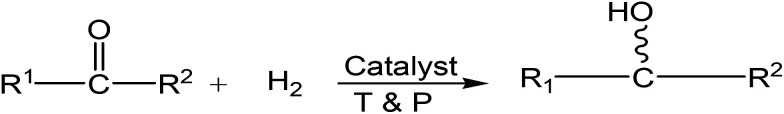


#### (IV) Hydrogenation–esterification

One-step hydrogenation–esterification is another upgrading process, in which bio-oils (derived from pyrolysis of biomass) are converted into biofuel or biochemicals (biolubricants). The final products are suitable for combustion and become static oxygenous hydrocarbons, as they are hydrogenated esters in which esterification and hydrogenation are the fundamental reactions. The main constituents of bio oil are fatty acids, aldehydes and ketones, and phenols, which have a negative effect on the properties of bio-oils. Acetic acid, levulinic acid, furfural, hydroxyacetone, phenol, and ethane diol are considered as model compounds for producing biolubricants. To convert these mixtures into combustible and stable compounds (*i.e.* esters), a one-step hydrogenation–esterification (OHE) process is performed over different bifunctional, RANEY® Ni (RNs) or Lewis acid catalysts in methanol/ethanol.
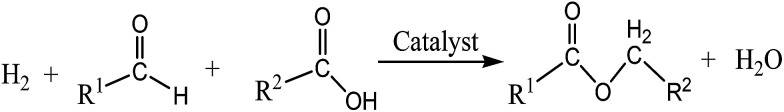


### 2.2 Properties of synthetic biolubricants

#### Viscosity index

Viscosity index (VI) indicates the variation of viscosity with temperature. A high viscosity index reveals small changes in viscosity with temperature. Biomass ester-based lubricants have a higher VI than conventional petroleum oils, which ensure that biolubricants retain the same activity in a wide range of temperatures by maintaining the oil film.

#### Pour point

It determines the temperature below which oils cannot be used as a lubricant in the engine. Pour point is an important factor for any lubricating oil. Biomass-derived ester-based lubricants have lower pour points than mineral oils, thus providing excellent lubrication in cold conditions.

#### Flash point

Lubricants have to tolerate high temperature when they are used. A flash point is the lowest temperature at which a lubricant must be heated when using before it vaporises. When mixed with air, a lubricant will flame up but will not burn. Flash points indicate lubricant volatility and fire-resistance properties, which are important factors for transportation. Biomass based-biolubricants have higher flash points than mineral oils.

#### Oxidation stability

Lubricants are generally oxidised to form various compounds. Oxidation stability of lubricants indicates the ability of resistance towards formation of oxides and sulphides, which increases when temperature rises. The most important things that lead to oxidation are temperature, pressure, metal surfaces, agitation, water, and contaminants. A low oxidative stability indicates that oil oxidises rapidly upon use if it is untreated. Biomass-derived ester-based lubricants have a higher oxidation stability than mineral oils.

#### Biodegradability

Biodegradability measures the ability of a material to be decomposed by microorganisms. A lubricant is classified as biodegradable if its degradation rate in a standard test exceeds a targeted level. Biomass-derived oils exhibit better biodegradability, of about 90–99%,^27^ whereas mineral oil biodegradability is about 20–40%. Biodegradability of lubricants is mainly influenced by the main component present in the base oil; hence, it depends on the structures of organic compounds.

### 2.3 Industrial application of biolubricants

**Fig. 2:**
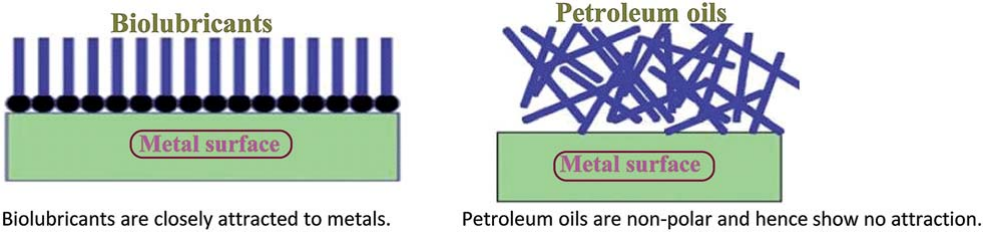
Bio-based lubricants possess a polar attraction to metals; by contrast, petroleum-based fluids have no polarity and no affinity to metals.

Ester-based biolubricants have been used as alternative lubricants in industries and automotives as they reduce friction and wear and operating noise and improve heat transfer. They have a longer adherence period on metal surfaces, as they have polar-based chemical structures, whereas petroleum-based lubricants are non-polar hydrocarbons and do not exhibit adhering properties on metal surfaces (Fig. 2). The application of biolubricant products in automotive industry are including hydraulic fluids, metal working oils, two-stroke and three stroke engine oils,^37^ and chainsaw fluids.^38^ In addition, biolubricants are extensively used as engine oils, transmission fluids, gear box oils, and brake and hydraulic fluids and are able to provide a slippery surface, reduce metallic corrosion and enhance performance of the machinery (Table 5).

**Table 5:** Specific application sectors of biolubricants^39,40^

Maintenance areas	Specific applications
In automotives	Engine oils, brake fluids, gear oils, gasoline engine oils, greases
In aviation	Turbine fluids, hydraulic fluids, piston engine fluids, lubricating greases
In industry	Gas turbine fluids, hydraulic oils, circulation and bearing oils, air compressor fluids, gas compressor oils, metal working fluids, lubricating greases and heat transfer oils

### 2.4 Advantages of biolubricants

The benefits of biolubricants are that they produce a cleaner, safer, less toxic environment and cause fewer skin problems for those who are working with engines and hydraulic systems. The physical and chemical properties of biolubricants are safer owing to their higher flash point, low pour point, constant viscosity, and less vapor emissions and oil mist.^41^ Moreover, they produce a low emission due to the high range of boiling temperature of esters. They can reduce pollution in stormwater from leaks in engines, hydraulic systems, and brake lines. These biolubricants are highly biodegradable^42^ and their cost is less over the product's life-cycle, as less maintenance^43^ and storage and disposal systems are needed. However, they are 3–5 times more expensive than petroleum oils (Table 6).^44^

**Table 6:** Comparison for physicochemical properties of biolubricants and petroleum oils^27,33,45^

Properties	Standard test method	Biolubricant	Petroleum oil
Density at 20 °C (kg m^−3^)	ASTM D445-15a	930–950	880
Viscosity index (VI)	ASTM D 445	150–200	100
Pour point, °C	ASTM D97-12	−20	−15
Flash point	ASTM D92-12b	Good	Poor
Cold flow behaviour	ASTM D5949	Good	Poor
Oiliness	ASTM D6079	Good	Poor
Miscibility with petroleum oils	ASTM 17025	Good	Poor
Oxidation stability	ASTM D2440	Moderate	Good
Biodegradability	EN 45000	Good	Poor
Sludge forming affinity	ASTM D2070	Poor	Good
Price, Euro per L	—	3–5	1

## 3 Catalytic reaction for synthesis of biolubricants

The homogeneous catalysts are molecularly dispersed in the reacting fluids; hence, pore diffusion limitations are absent and bulk phase mass transfer limitations usually occur. Homogenous catalytic reactions are used not only for the biolubricant production^46,47^ but also for many conventional organic transformation reactions (Table 7).

**Table 7:** Homogeneous acid–base catalysts for ester based-biolubricant synthesis^54,59,69^

Type of catalyst	Advantages	Disadvantages
**Homogeneous catalyst**		
*Acid catalyst*		
Liquid acid, *e.g.* H_2_SO_4_, HCl, H_3_PO_4_	Easily react with fatty acids and alcohols, forming ester based-biolubricants	Product neutralization and separation problem due to corrosive nature of these acids
Metal salt (Lewis acid), *e.g.* Sn, Cu, Co, Ni salts	Easily react with feedstock and alcohols in solution, giving better yield	Catalyst separation from product is difficult due to high solubility in the solution
Ligand metal complex (Lewis acid), *e.g.* Sn, Co, Ni, Fe, Ru complexes	Molecularly dispersed in the reacting fluids, giving better yield with high product selectivity. Hence, reaction mechanism and kinetics are easy to understand	Catalyst separation is difficult and requires high technology, which is sometimes expensive

*Base catalyst*		
Schiff base complex (Lewis base), *e.g.* Co, Ni, Cd, Zn complexes	Transesterification and hydrogenation reactions are influenced by these catalysts	As they are a little basic and soluble in reacting fluids, they are used in the presence of another base

The transition metal complexes (present in homogeneous form) are acidic and thus potentially catalyse many organic reactions (*e.g.* esterification, transesterification, and ester hydrolysis). Transition metal complexes are potentially applied as acid catalysts for ester-based lubricant synthesis. It exhibits properties of chemoselectivity, regioselectivity,^48^ and enantioselectivity in which the reaction mechanism and kinetics are easy to understand. On the other hand, heterogenous catalyst are poorly defined in the reacting fluids, and their synthesis problem and reaction mechanism are rarely understood. In case of heterogeneous catalysts, the reaction mixture is not well dispersed because of their larger particle, so a low yield with low selectivity is usually observed. In this context, as a homogeneous catalyst is well dispersed within the reacting fluid, it is chosen for the production of biolubricants. In fact, transition metal complexes in the homogeneous form are reviewed for further study. Table 13 briefly illustrates these catalysts.

### 3.1 Catalytic esterification reaction

Ester-based biolubricants are generally prepared by the esterification reaction between long-chain carboxylic acids (C_4_–C_14_) (*e.g.* butanoic acid, pentanoic acid, levulinic acid, oleic acid and linoleic acid)^49^ with polyols including glycerol (Gly), neopentyl glycol (NPG), trimethylolpropane (TMP), pentaerythritol (PE) and *n*-octanol, and *n*-butanol in the presence of liquid acid (*e.g.* H_2_SO_4_, HCl, HNO_3_) or Lewis acid catalysts (*e.g.* metal salts or transition metal complexes). Basically, the liquid acid catalyst used for the esterification reaction causes major corrosion of the reacting system and produces a salt as a result of the neutralisation reaction with base chemicals. Besides, additional cost is required for product washing and water separation from the final liquid ester product.^50^ In this part, a comparative study on the homogeneous^51^ and heterogeneous^52^ catalysed esterification reactions are reviewed herein.

Noor *et al.*^53^ studied the catalytic performance of perchloric acid, sulfuric acid, hydrochloric acid, and nitric acid (liquid acid) for biolubricant production. The process involved the esterification between *Jatropha curcas* oil with trimethylolpropane (TMP) at 150 °C, reaction time of 3 h and molar ratio of FA : TMP was 4 : 1 with 2% w/w concentrated catalyst. They revealed a higher TMP ester yield, which was dependent on the esterification catalysts's acidity strength; here perchloric acid had the strongest acidity, giving a maximum of 70% TMP–ester. By contrast, sulfuric acid, *p*-toluenesulfonic acid, hydrochloric acid and nitric acid gave 46%, 42%, 41% and 37% ester yield, respectively.

**Fig. 3:**
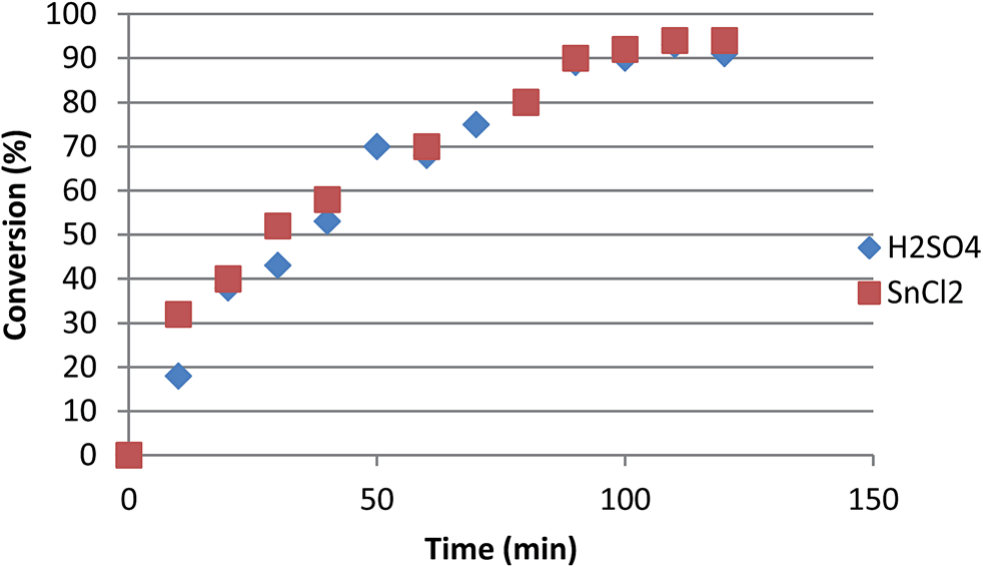
Conversion of oleic acid alcoholysis with ethanol catalyzed by H_2_SO_4_ and SnCl_2_.^54^

Abiney *et al.*^54^ assessed the influence of SnCl_2_ as the catalyst on the conversion of oleic acid to ethyl oleate in C_2_H_5_OH solution; the oleic acid : catalyst ratio was 100 : 1 (v/w), under 80 °C of temperature for 6 h reflux. The use of SnCl_2_ has more advantages than the use of mineral acid catalysts, as the former is less corrosive, less expensive, shows Lewis acid characteristics, is required in low amounts, and is able to avoid unnecessary neutralisation of the products. The SnCl_2_ has shown a better catalytic activity than mineral H_2_SO_4_ acid and a better conversion rate with high selectivity (Fig. 3).

**Fig. 4:**
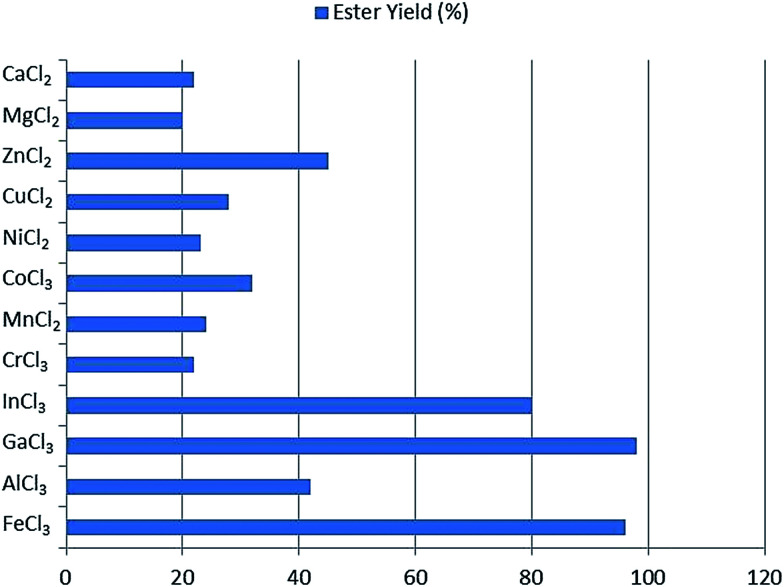
Catalytic activities of various metal chlorides for esterification of palmitic acid with cetyl alcohol.^55^

Ieda *et al.*^55^ studied the catalytic performance of a series of metal salts for the esterification process of fatty acids with alcohols, as shown in Fig. 4. Metal salts have Lewis acid character and are highly soluble in reacting fluids, and are used as the catalyst for the esterification reaction. Long-chain fatty acids including C_10_–C_18_ and alcohols were examined in equimolar ratios in the catalysts, substrate/catalyst = 200 (v/w) and reaction period = 6 h in mesitylene solution. The salts including chlorides, sulfates, nitrates and acetates of Al^3+^, Fe^3+^, In^3+^, ZrO^2+^, HfO^2+^, Zn^2+^, Ni^2+^, Co^2+^, Cr^3+^, Cu^2+^, Mg^2+^ and Mn^3+^ were used. The conversion rate and selectivity depended on the amount of the catalyst used and the reaction time. Ferric salts, particularly, FeCl_3_·6H_2_O was the most active and 90% yield was attained in this case.

**Fig. 5:**
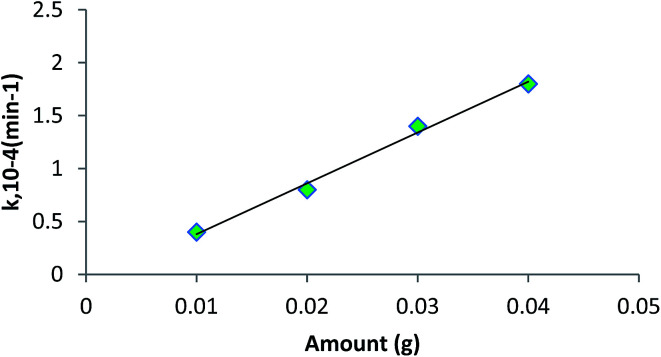
Plot of rate constant (*k*) *vs.* amount of catalyst loading; [Co(Q)(Val)]·H_2_O.^56^

Shivankar *et al.*^56^ synthesised the Co(ii) and Ni(ii) mixed ligand complexes using 8-hydroxyquinoline (Q) as the primary and amino acids (HA) as secondary ligands for ester hydrolysis. The hydrolysis of ethyl acetate and methyl acetate was studied using metal complexes as homogeneous catalysts. The amount of ester was produced under a reaction temperature of 40–50 °C) and substrate/catalyst ratio = 100–500 (v/w) to study the reaction mechanism and kinetics. The reaction mixture (5 mL) was withdrawn at regular intervals for titration against standard NaOH solution to measure the acid values. It was evident from their experiment that mixed ligand metal complexes are more efficient catalysts for ester hydrolysis. The rate constant (*k*) value increased (2) with an increase in the amount of the catalyst (0.05 g) at 40 °C and ethyl acetate was 5.0 cm^3^ as shown in Fig. 5.

The plot between *k* and the amount of catalyst (Fig. 5) used is a straight line, showing that the rate constant (*k*) is proportional to the amount of catalyst used for the hydrolysis process.

Shivankar *et al.*^57^ in another report investigated the chiral mixed ligand complexes of 8-hydroxyquinoline as primary ligands, while dextrose, fructose, mannitol and tartaric acid were secondary ligands for ester hydrolysis. The hydrolysis of ethyl acetate and methyl acetate was studied using metal complexes as the homogeneous catalysts. The hydrolysis of ester is the opposite of the esterification reaction and is catalysed by transition metal complexes; researchers would like to determine the change in enthalpy (Δ*H*), entropy (Δ*S*) and Gibbs free energy (Δ*G*) in an activated state. In this case, the amount of ester at a temperature of 30–50 °C was being constant when the amount of catalysts were varied, with substrate/catalyst = 100–500 (v/w). Table 8 summarises that the rate constants are highly dependent on the type of catalyst at constant temperature (40 °C), where changes in Gibb's free energy was almost similar, while the reaction entropy largely varied in an activated state. The value of rate constant (*k*) increased with the type of catalyst used; [Co(Q)(Val)]·H_2_O was found to have obtained the highest kinetic constant. The amount of catalyst was strongly dependent on the kinetic constant summarised in Table 8. For instance, [Co(Q)(Val)]·H_2_O consumed the least amount of catalyst as the rate constant was directly related to the amount of catalyst involved in the reaction.

**Table 8:** Comparison of kinetic hydrolysis of ethyl acetate in the presence of complexes as catalysts; Q = 8-hydroxyquinoline, Dex = dextrose, Fru = fructrose, Man = manitol, Val = l-valine, Phe = l-phenylalanine

Complex	*T*, (°C)	*k* × 10^−2^, (min^−1^)	Δ*H*^#^, (kJ mol^−1^)	Δ*S*^#^, (kJ mol^−1^)	Δ*G*^#^, (kJ mol^−1^)
[Co(Q)(Val)]·H_2_O	40	1.64	36.62	−226.30	107.48
[Co(Q)(Man)]·H_2_O	40	1.11	48.46	−191.35	108.35
[Co(Q)(Phe)]·H_2_O	40	1.27	47.54	−192.2	107.69
[Co(Q)(Fru)]·H_2_O	40	1.45	50.16	−183.20	107.50
[Ni(Q)(Phe)]·H_2_O	40	0.69	30.81	−248.09	109.19
[Ni(Q)(Dex)]·H_2_O	40	0.79	32.89	−244.09	109.29

**Fig. 6:**
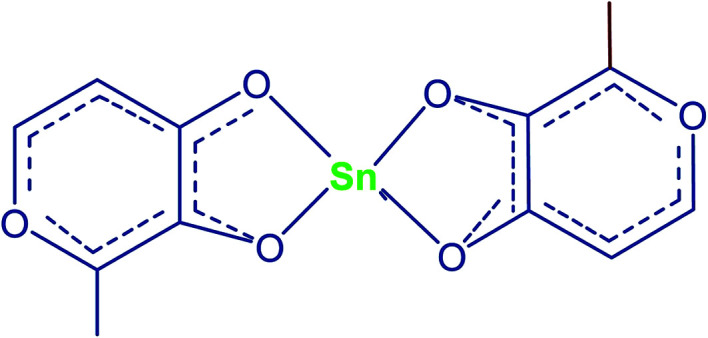
Chelate structure of tin-HMP.^51^

Oliveira *et al.*^51^ carried out an experiment using bivalent tin chelate of 3-hydroxy-2-methyl-4-pyrone (HMP) complex (Fig. 6) as a homogeneous catalyst for poly-esterification of neopentyl glycol (NPG), terephthalic acid (TFA) and trimethylolpropane (TMP) by using acid : alcohol ratio of 4.8 : 4.2 : 2.1 at 150–230 °C for 5 h reflux. The Sn(iv) with Lewis acid character together with its chelate complexes can enhance the esterification reaction by expanding the metal's d orbitals to increase the accessibility towards substract molecules *via* an associative exchange with ligands. By increasing the amount of catalyst loading, they observed a decrease in the reaction time, although the productivity was higher for reactions with lower catalyst loading. So, compared with other properties, they concluded that the behaviour was not linear (Table 9).

**Table 9:** Main results for polyesterification using different amounts of tin catalysts^51^

Entry no.	Substrate/catalyst ratio (v/w)	Reaction time (h)	Viscosity (CP)	Productivity	Water (g)
01	100	18.31	485	364.40	142.29
02	200	13.25	355	250.80	163.93
03	300	13.20	133	235.00	131.58
04	400	11.30	330	154.80	161.03
05	500	12.55	525	104.30	146.44
06	600	9.35	385	117.00	170.22

**Fig. 7:**
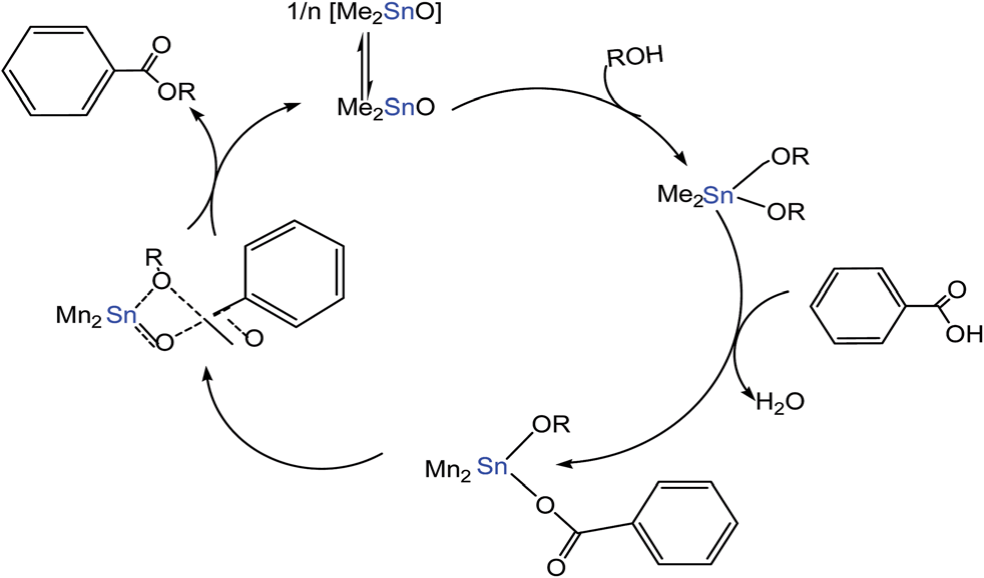
Schematic pattern of organotin(iv)-based catalyst for esterification using Me_2_SnO as a catalyst.^58^

Meneghetti *et al.*^58^ reported on Sn(iv)-based complexes as catalysts to produce alkyl esters from alcohol and carboxylic acid. The researchers proposed two types of reaction mechanisms; Lewis acid and exchange/insertion mechanism by introducing labile ligands, which can be exchanged between the feedstock and catalyst (Fig. 7). The Sn metal having vacant 5d orbitals could expand its coordination numbers by insertion with subtract molecules through non-bonding electron pairs and enhance the esterification reactions. First, Sn(iv) complexes bonded with alcohol to form an intermediate product. This intermediate product was then reacted with carboxylic acid, forming another transition compound with the ester product and water as by-products.

**Fig. 8:**
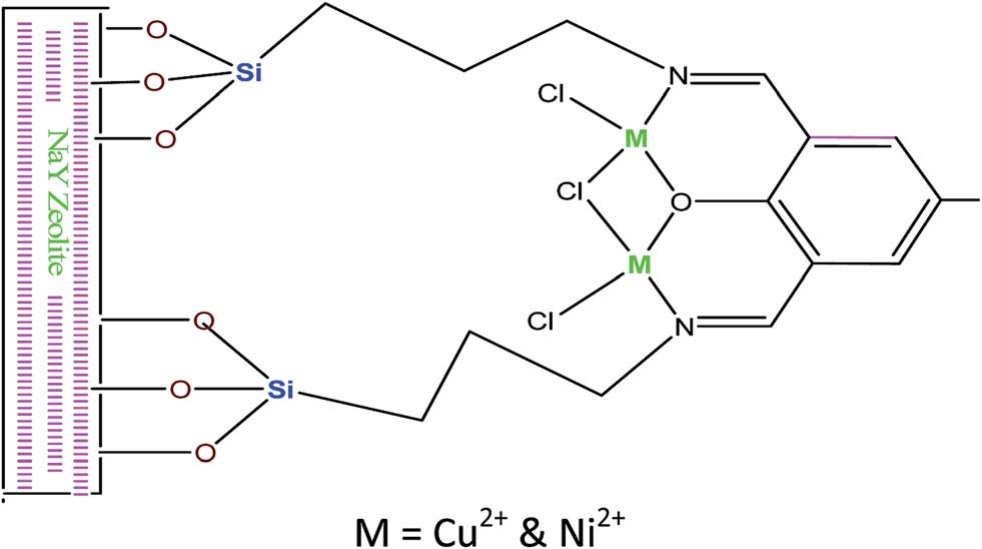
Illustration pattern of immobilization DFP and related complexes.^59^

Zendehdel *et al.*^59^ reported that the utilisation of NaY zeolite supported 2,6-diformyl-4-methylphenol (DFP) complexes (Fig. 8) as the heterogeneous organic catalyst for esterification of acetic acid with different alcohols (*e.g.* 2-pentanol, isoamyl alcohol). The results indicated that the Schiff based complexes^60^ had a better catalytic activity towards esterification. The NaY zeolite catalyst supports the Schiff base complexes with available of Na ions on the zeolite Y matrix, which gives NaY–NH_2_ a strong basic character even without organic bases. Hence, the fixation of Schiff-based complexes over the NaY–zeolite surface could enhance the catalyst activity^61^ towards the esterification reactions. The esterification was carried out at 70 °C for 2 h with 50 mg of catalyst loading and a maximum of 90% conversion of acetic acid.

Oh *et al.*^62^ reported on the preparation of biolubricants using long-chain alcohols (8 carbon atoms or more) with saturated and unsaturated fatty acids over sulphated zirconia as the potential heterogeneous catalyst.^63^ Sulphated zirconia is a solid acid catalyst for developing environmentally benign and friendly processes for esterification, as they have acidic sites and the so-called solid superacid catalyst. Sulphated zirconia compounds consist of monoclinic and tetragonal zirconia phases, and the transition from the monoclinic phase to the tetragonal of zirconia is attributed to sulfate content in the Zr–O–S framework. The incorporation of SO_4_^2−^ into the ZrO_2_ matrix is achieved by the wet impregnation method by varying the SO_4_^2−^ content. The alcohol structure is found to have greatly affected the conversion rate and the product yield. The yield of the product and rate of conversion for the esterification of stearic acid and unsaturated acids (*e.g.*, oleic acid, linolenic acid and linoleic acid) with various alcohols using 6.25 mmol fatty acid, 7.5 mmol alcohol and 100 mg of catalyst at 140 °C for 4 hours is shown in Table 10.

**Table 10:** Esterification of several alcohols with fatty acids and sulphated zirconia as catalyst, OcOH = octanol^62^

Alcohols	Free fatty acids (FFA)	Conversion of FFA (%)	Yield of product (%)
1-OcOH	Stearic acid (StA)	97.80	93.90
1-OcOH	Oleic acid (OA)	90.40	88.60
1-OcOH	Linolenic acid (LinOA)	86.30	84.60
Tetradecanol	Oleic acid (OA)	87.30	83.20
Hexadecanol	Oleic acid (OA)	85.70	81.70
2-OcOH	Oleic acid (OA)	85.20	82.20
3-OcOH	Oleic acid (OA)	31.00	28.10

Adam *et al.*^64^ described the kinetic study for the esterification of ethyl alcohols and acetic acid over the heterogeneous l-phenylalanine–Ru(iii) complex immobilisation on silica. Initially, the reaction was performed with 0.10 g catalyst using ethyl alcohol to acetic acid ratio of 1 : 3 at 85 °C and the conversion of ethyl alcohol in 1 h was 34% and the conversion reached 94% over the next 12 hours. As silica was grafted on Ru(iii) site, the complex catalyst rendered an acidic character capable of enhancing the esterification reaction. This catalyst could be regenerated by washing with ethanol and further be used without significant loss of reactivity.

Kotwal *et al.*^18^ reported that the biolubricants were synthesised using three-dimensional compounds of titanosilicates, Ti-SBA-12 and Ti-SBA-16. The incorporation of Ti in the silica framework produced Lewis acid sites that were active for the esterification reaction. The higher catalytic performances of these catalysts were due to Lewis acidic Ti sites and the mesoporous character of their structure. In a typical reaction containing 0.12 g catalyst, the oleic acid to polyol (NPG, TMP and PE) ratio was 1 : 1 and reaction temperature was 180 °C with 1 h continuous reflux condition; the following results of these complexes were found (Table 11).

**Table 11:** Catalytic activities of Ti-SBA-12 & Ti-SBA-16 for esterification; Ti-SBA-12 = 40, Ti-SBA-16 = 50 ^18^

Polyolacs	Catalysts	OA conversion (mol%)	Ester selectivity (mol%)		
			Mono	Di	Tri
Trimethylolpropane (TMP)	Ti-SBA-12	75.40	62.80	36.20	1.10
	Ti-SBA-16	71.10	70.20	28.10	1.50
Neopentyl glycol (NPG)	Ti-SBA-12	52.40	87.90	12.10	—
	Ti-SBA-16	62.70	86.60	13.40	—
Pentaerythritol (PE)	Ti-SBA-12	36.60	72.60	18.40	9.00
	Ti-SBA-16	31.10	77.60	18.80	3.60

Maki *et al.*^65^ compared the catalytic activity of *N*-alkyl-4-boronopyridinium halides and boric acid (H_3_BO_3_) *via* esterification of α-hydroxycarboxylic acids with different alcohols (methanol, ethanol, propanol, butanol). The results suggested that *N*-methyl-4-boronopyridinium iodide showed a better reactivity than H_3_BO_3_ catalyst with a maximum yield of 90%, which was attained for 6 h reflux. However, boric acid catalyst was more effective for dehydrating esterification between an equimolar ratio of α-hydroxycarboxylic acids and alcohols. *N*-Polystyrene bound with 4-boronopyridinium chloride to form a heterogeneous catalyst was also effective and could be reused after filtration.

Nandiwale *et al.*^66^ reported the production of octyl levulinate biolubricants by the esterification process over the modified H-ZSM-5 (Meso-HZ-5) catalyst (where Si/Al ratio = 37) of biomass-derived levulinic acid with *n*-octanol and achieved a 99% yield at 100–120 °C with 10–30 wt% (LA) catalyst. This was an efficient catalytic process for conversion of agricultural waste feedstock to valuable chemicals. The ZSM-5 zeolite is composed of several pentasil units linked together by oxygen bridges to form a pentasil chains and is a medium-pore zeolite with channels defined by ten-membered rings; H-ZSM-5 is in protonated form. The ZSM-5 zeolite catalyst exhibits Lewis acidity that enhances the esterification reaction (Table 12).

**Table 12:** Summary of Lewis acid catalyst for esterification reaction

Catalyst	Catalyst type	Specific catalyst	Reaction conditions	Feedstock	Conv. (%)	Yield (%)	Ref.
Homogeneous	3.1 Liquid acid	HClO_4_, H_2_SO_4_, HCl, HNO_3_	*T* = 150 °C, 2 w/w catalyst at 3 h reflux	*Jatropha curcas* oil	80	70	53
		H_3_BO_3_	Alcohol : acid = 1 : 1, catalyst = 5–10 mol% with reflux	α-Hydroxy carboxylic acids	90	90	65
	3.2 Metal salt	(1) SnCl_2_	(1) Substrate/catalyst = 120, SnCl_2_ = 0.01–0.4 mmol followed by reflux	Oleic acid	95	90	54
		(2) FeCl_3_	(2) Substrate/catalyst = 200, reflux in mesitylene, time = 6 h	Stearic, myristic, and capric acids	90	95	55
		(3) ZrOCl_2_	(3) Substrate/catalyst = 1, 5 mol% salt, *T* = 50 °C, time = 24 h	Acrylic acids, carboxylic acids	80	75	67
	3.3 Metal complex	(1) Co(ii), Ni(ii) complexes	Ester in DMF, catalyst = 0.01–0.04 g, *T* = 30, 40 and 50 °C	Methyl acetate and ethyl acetate	90	89	56
		(2) Co(ii), Ni(ii) chiral complexes	Ester in DMF, catalyst = 0.01–0.04 g, *T* = 30, 40 and 50 °C	Methyl acetate and ethyl acetate	85	90	57
		(3) Tin chelate, Sn(C_6_H_5_O_3_)_2_ complex	TFA, NPG and TMP are 4.8 : 4.2 : 2.1, *T* = 150–230 °C, time = 5 h	Terephthalic acid (TFA)	83	78	51
		(4) Sn(iv) organometallic complexes	MeOH/EtOH : oil : catalyst = 400 : 100 : 1; *T* = 80 °C; stirring = 1000 rpm; OG reactor	Soybean oils	89	75	58
Heterogeneous	3.4 Supported metal complex	(1) Cu(ii), Ni(ii) complex with zeolite support	Catalyst = 50 mg, CH_3_COOH = 50 mmol, alcohol = 100 mmol, *T* = 70 °C, time = 2 h	Acetic acid	60–92	93	59
		(2) Sulphated zirconia, Zr(OCH_2_, CH_2_CH_3_)_4_	Fatty acid = 6.25 mmol, alcohol = 7.5 mmol, catalyst = 100 mg, *T* = 140 °C, time = 4 h, stirring = 300 rpm	Oleic acid	90	84	62
		(3) Ru(iii) complex on silica support	Ethanol = 0.20 mol, catalyst = 0.10 g, acetic acid = 0.20 mol, *T* = 85 °C, time = 9 h	Acetic acid	80	94	64
		(4) Ti-SBA-12 & Ti-SBA-16, titanosilicates	OA : polyol = 3 : 1 and 1 : 4, catalyst = 3 wt% of OA, *T* = 180 °C, time = 1–10 h	Oleic acid	82	92	18
		(5) Modified H-ZSM-5	*n*-Octanol : LA molar = 4–10, catalyst = 10–30 wt% (LA), *T* = 100–120 °C, time = 5 h	Levulinic acid	90	99	66

### 3.2 Transesterification reaction

Transesterification pathway is another conventional process to synthesise ester-based biolubricants using triglyceride-based feedstock and alcohol with the aid of a catalyst. Transesterification is more advantageous compared to general acid-catalysed esterification processes from carboxylic acids and alcohols. For example, some carboxylic acids are sparingly soluble in organic solvents and homogenise esterification with great difficulty, while esters are generally soluble in most organic solvents. The ester-to-ester transformation is highly suitable when the parent carboxylic acids are changeable and difficult to isolate. The most frequently used acid catalysts are sulfuric, sulfonic, phosphoric, and hydrochloric acids. Base-catalysed transesterification is another conventional method, and a wide variety of metal alkoxides, acetates, oxides, and carbonates work in this method. The most popular bases are sodium and potassium alkoxides. Acid catalysed transesterification is much slower than alkali catalysed transesterification and typically requires a higher temperature. However, acid-catalysed transesterifications are more favourable than base catalysed transesterifications, since acid catalysis is not highly affected by the presence of free fatty acids in the feedstock. Practically, acid catalysts can simultaneously catalyse both the esterification and transesterification reactions. The most commonly used alcohol for this purpose is methanol and the process is sometimes called methanolysis. Here we intend to describe the Lewis acidity of catalysts that enhance the transesterification reaction. For this, we include some metal salts, especially Sn(ii) and Sn(iv) based complexes with different ligands, which are reviewed. The main advantages of these catalysts are that they are highly soluble in the oil phase where the reaction occurs and give the maximum yield, but catalyst separation is quite difficult.

Hao *et al.*^68^ utilised Sn(iv), Hf(iv) and Yb(iii) bis(perfluorooctanesulfonyl) amide complexes in the transesterification reaction between methyl butyrate (MB) and *n*-octanol (*n*-OcOH). The catalytic activity for transesterification was strongly affected by the Lewis acid character of the catalyst complexes. The findings revealed that Sn[N(SO_2_C_8_F_17_)_2_]_4_ catalyst exhibits an excellent yield of 89% with 99% selectivity for transesterification at an equimolar ratio of methyl butyrate with *n*-octanol in a fluorous biphase system (FBS). All the catalytic activity of Sn(iv), Hf(iv) and Yb(iii) was evaluated using a fluorous solvent, which enhances the immobilisation of the catalyst and phase separation at the end of reaction (also known as FBS system). Table 13 shows the catalytic performances of metal complex catalysts, which were investigated in the FBS system under 80 °C for 15 h. It was revealed that Sn[N(SO_2_C_8_F_17_)_2_]_4_ was the outperformed catalyst due to the catalyst being completely immobilised in the fluorous phase for the transesterification and the fluorous solution being directly used in the subsequent reaction, *i.e.*, it being recyclable.

**Table 13:** Catalytic transesterification reaction between methyl butyrate and *n*-octanol in fluorous biphase system (FBS)^68^^,^^a^

Entry no.	Catalysts	Yield (%)
1	Sn[N(SO_2_C_8_F_17_)_2_]_4_	89
2	Sn[N(SO_2_C_8_F_17_)_2_]_2_	84
3	Sn(OSO_2_CF_3_)_2_	83
4	Hf[N(SO_2_C_8_F_17_)_2_]_4_	76
5	Hf(OSO_2_CF_3_)_4_	65
6	Yb(OSO_2_CF_3_)_3_	12

a Results of conversions are not provided by the study.

Abreu *et al.*^69^ tested three homogeneous catalysts, (i) Sn(HMP)_2_(H_2_O)_2_, (ii) Pb(HMP)_2_(H_2_O)_2_, and (iii) Zn(HMP)_2_(H_2_O)_2_ (HMP = 3-hydroxy-2-methyl-4-pyrone) for the transesterification reaction of triglycerides with methanol. Andiroba, babassu, piqui, cumaru, palm and soybean oil methanolysis were performed at 60 °C with a reaction condition of methanol : oil : catalyst ratio of 400 : 100 : 1 (v/v/w) with a reaction time of 1 h. Table 14 depicts the summary of catalytic performances of Sn-, Pb- and Zn-based complexes, and the H_2_SO_4_ catalyst. It has been concluded in the study that catalytic activity decreases in the order Sn^2+^ ≫ Zn^2+^ ≫ Pb^2+^ with decreasing Lewis acid character. Sn has a stronger Lewis acid character due to it having vacant 5d orbitals that can expand its coordination numbers by insertion with subtract molecules, which gives a higher yield.

**Table 14:** Methanolysis of various vegetable oils catalyzed by Sn-, Pb- and Zn-based complexes and H_2_SO_4_ (ref. 70)^a^

Vegetable oils	Catalysts	Yield (%)	Composition of fatty acids	
			Unsaturation (%)	Chain size (% C)
Soybean	H_2_SO_4_	1.40	76	14
	Sn(HMP)_2_(H_2_O)_2_	37.10		
	Pb(HMP)_2_(H_2_O)_2_	4.20		
	Zn(HMP)_2_(H_2_O)_2_	15.50		
Andiroba	H_2_SO_4_	3.80	66	28
	Sn(HMP)_2_(H_2_O)_2_	23.30		
	Pb(HMP)_2_(H_2_O)_2_	5.20		
	Zn(HMP)_2_(H_2_O)_2_	11.20		
Palm	H_2_SO_4_	8.50	58	35
	Sn(HMP)_2_(H_2_O)_2_	16.20		
	Pb(HMP)_2_(H_2_O)_2_	5.40		
	Zn(HMP)_2_(H_2_O)_2_	11.30		

a Results of conversions are not provided by the study.

Moreover, Serra *et al.*^71^ also reported on the methanolysis of castor oil and soybean oil using homogeneous Sn(iv)-based catalysts. The Sn(iv)-based catalysts are dibutyltin diacetate ((C_4_H_9_)_2_Sn(C_2_H_3_O_2_)_2_), di-*n*-butyl-oxo-stannane ((C_4_H_9_)_2_SnO), butylstannoic acid ((C_4_H_9_)SnO(OH)) and dibutyltin dilaurate ((C_4_H_9_)_2_Sn(C_12_H_23_O_2_)_2_), which were taken as Lewis acid catalysts for transesterification reactions. Two types of reactors were used, open glass reactor (OG), where the reactions were performed under methanol reflux at atmospheric pressure (65 °C), and closed steel reactor (CS), where the reactions were carried out at 80 °C and 120 °C. The reaction conditions for both reactors were MeOH : oil : catalyst = 400 : 100 : 1 at constant magnetic stirring of 1000 rpm. The results implied that the methanolysis of the castor oil led to lower yields than soybean oil although Sn(iv) catalyst was employed due to the influence of the chemical composition of the triglycerides on the activity of the catalysts based on the Lewis acid sites. Another important observation was the use of a CS reactor rather than an OG reactor, which increased the reaction temperature, leading to greater reaction yields for all Sn(iv) complexes. The FAMEs (% yield) by methanolysis of castor and soybean oil in the presence of Sn(iv) catalysts, using OG and CS reactors, are depicted in Table 15. The (C_4_H_9_)_2_SnO catalyst gave the highest yield in the closed steel reactor because this was attained under high reaction temperature with high methanol concentration in the liquid phase, giving high reaction rates during methanolysis.

**Table 15:** FAMEs (%) yield produced from catalytic transesterification of castor and soybean oil with the presence of Sn(iv) complexes; (C_4_H_9_)_2_Sn(C_2_H_3_O_2_)_2_, (C_4_H_9_)_2_Sn(C_12_H_23_O_2_)_2_, and (C_4_H_9_)_2_SnO^71^

Reactor temperature (°C)	Reaction time (h)	(C_4_H_9_)_2_Sn(C_2_H_3_O_2_)_2_		(C_4_H_9_)_2_Sn(C_12_H_23_O_2_)_2_		(C_4_H_9_)_2_SnO	
		Soybean oil	Castor oil	Soybean oil	Castor oil	Soybean oil	Castor oil
OG	1	8	<5	7	<5	<5	<5
	2	13	<5	11	<5	<5	6
	4	23	<5	20	<5	7	<5
CS 80 °C	1	32	<5	47	6	35	<5
	2	63	<5	48	7	48	12
	4	75	<5	62	8	64	16
CS 120 °C	1	56	28	70	19	45	8
	2	73	47	77	23	83	23
	4	77	64	76	36	85	46

Ferreira *et al.*^72^ investigated the methanolysis of soybean oil in the presence of tin(iv) complexes including FASCAT® 4100, 4201, and 4350 and LIOCAT® 118 catalysts (Table 16) under mild conditions at 80 °C, methanol : oil : catalyst ratio of 400 : 100 : 1 (v/v/w), and reaction time of 10 h. Results indicated that the dibutyltin dilaurate catalyst offered the best reactivity in terms of reaction yield, approximately 43% of FAMEs after a 10 h reaction period.

**Table 16:** Chemical profile for commercial tin(iv) complexes

Catalysts	Chemical formula	Commercial name	Yield (%)
FASCAT® 4100	(C_4_H_9_)SnO(OH)	Butylstannoic acid	7
FASCAT® 4201	(C_4_H_9_)_2_SnO (modified)	Di-*n*-butyl-oxo-stannane	19
FASCAT® 4350	(C_4_H_9_)_2_SnO (98%)	Stannane (98%)	14
LIOCAT® 118	(C_4_H_9_)_2_Sn(C_12_H_23_O_2_)_2_	Dibutyltin dilaurate	43

**Fig. 9:**
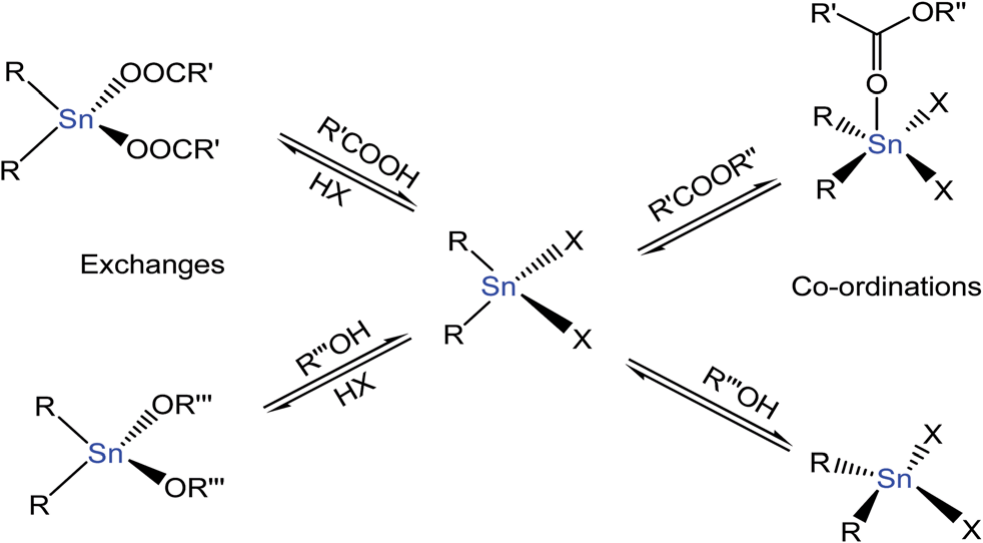
Coordination mode of additional ligands and associative exchange.^58^

The tin(iv)-based compounds have the potential to act as heterogeneous or homogeneous precursors for esterification, transesterification and/or polycondensation reactions. Meneghetti *et al.*^58^ proposed that the key role of organotin(iv) complexes for transesterification reactions was due to the Lewis acidity. Tin(iv) atoms can coordinate with many molecules in solution and associative exchanges in certain mobile ligands with other compounds. The proposed catalytic transesterification mechanisms involving organotin(iv) complexes including ligand association or exchange processes are shown in Fig. 9. In the case of exchange mechanism, Sn(iv) molecule were bonded with acid and alcohols to form an intermediate product. For coordination, Sn(iv) molecule was bonded with ester and alcohols to form a transition compound. In both cases, these products immediately decomposed, leading to ester and alcohols.

The transesterification reactivity of Sn(iv) compounds including Bu_2_Sn(Lau)_2_, BuSn(O)OH, and Bu_2_SnO was investigated by Meneghetti *et al.*^58^ In addition, the effect of the reaction parameters (reactor type, substrate to alcohol ratio, reaction time, temperature and catalyst loading) for the transesterification process is reported in Table 17. The finding reveals that the Bu_2_Sn(Lau)_2_ catalyst exhibits an excellent yield (98%) at 150 °C when MeOH : soybean oil : catalyst = 400 : 100 : 1 (v/v/w) with constant stirring at 1000 rpm. Bu_2_Sn(Lau)_2_ is the best catalyst compared to Bu_2_SnO and BuSn(O)OH due to its higher degree of solubility and activation, which can be reached at higher temperatures.

**Table 17:** FAMEs (% yield) through transesterification in the vicinity of BuSn(O)OH, Bu_2_Sn(Lau)_2_ and Bu_2_SnO catalysts in a closed steel reactor^73^

Reactor	Temperature (°C)	Time (h)	Catalysts		
			Bu_2_Sn(Lau)_2_	Bu_2_SnO	BuSn(O)OH
Closed steel reactor	80	1	47	35	—
		2	48	48	10
		4	—	64	—
	120	1	70	45	40
		2	77	83	76
		4	76	83	60
	150	1	98	75	70
		2	98	95	73
		4	80	74	74

Serio *et al.*^74^ reported that the most effective catalysts (*e.g.*, Cd, Mn, Zn, Pb carboxylic salts) have been individuated and a correlation of the activities with the cation acidity has been obtained. These catalysts are active in the presence of high FFA concentrations, whereas homogeneous alkaline catalysts pose great difficulties due to the presence of large amounts of free fatty acids (FFA). The study reveals that the catalysts activity depends on the metal acidity and the structures of ester and alcohols; hence, every ester–alcohol couple will have a specific metal choice that will give the maximum activity.^75^ Besides, these catalysts are more efficient in the presence of high free fatty acid concentrations with a 5 × 10^−3^ : 1 weight ratio of catalyst to oil at 200–250 °C when 2.0 g (0.2% w/w of FFA) of soybean oil and 0.88 g of methanol is used (Table 18). The results show that the Cd(OAc)_2_ catalyst successfully achieved a maximum of 89% conversion contrary to that of Ba(OAc)_2_, which generated a lower conversion under the same reaction conditions. Cd(OAc)_2_ is the most promising catalyst in this study due to it's resistant to deactivation during by-product water formation from esterification of FFA process. It is possible to obtain high FAME yields (96%) and a low final FFA concentration (<1%) in a relatively short reaction time (200 min) and low catalyst concentration (4 × 10^−4^ : 1 weight ratio of catalyst to oil).

**Table 18:** List of experiments carried out using acetates catalyst at 200 °C, Ac = acetate^74^

Entry	Triglycerides	Alcohols	Catalysts	Conversion (%)	Yield (%)
01	Soybean oil	Methanol	Ba(OAc)_2_	73	78
02			Ca(OAc)_2_	73	82
03			Mg(OAc)_2_·4H_2_O	72	73
04			Cd(OAc)_2_	89	96
05			Mn(OAc)_2_	62	68
06			Ni(OAc)_2_·4H_2_O	66	75
07			Co(OAc)_2_·4H_2_O	81	85

The most recommended and best catalyst for transesterification reaction from the above study with respect to economic prospects, reaction conditions and the conversion rate are Sn(ii) and Sn(iv) complexes. Since Sn has a vacant 5d orbital, showing a Lewis acid character, it is the most promising catalyst in this study due to it being lowered by the water formation during transesterification of FFA using a low amount of catalyst. The main advantage of Sn-based complex catalyst is that it is highly soluble in the oil phase. Tin(iv) atoms can coordinate with many molecules in solution and be associatively exchanged in certain mobile ligands with other compounds. They are cheaper, easily available, form complexes with other electron donating groups, and are useful for biolubricant production through transesterification process. In fact, transesterification is ester-to-ester transformation, and the most commonly used alcohol is methanol and hence transesterification is sometimes called methanolysis (Table 19).

**Table 19:** Summary of Lewis acid catalyst for transesterification reaction

Catalyst type	Specific catalyst	Reaction conditions	Feedstocks	Conv. (%)	Yield (%)	Ref.
Metal complex	Sn(iv), Hf(iv)	Ester/alcohol = 1, catalyst = 0.05 mmol, reflux, *T* = 50 °C, time = 8 h	Methyl butyrate	90	89	68
	Sn(ii), Pb(ii), Zn(ii)	Alcohol : vegetable oil : catalyst = 400 : 100 : 1, and reflux at *T* = 60 °C, time = 1 h	Soybean, babassu, piqui, palm oils	85	90	69
	DBTDA and DBTDL	Methanol : oil : catalyst = 400 : 100 : 1 with stirring = 1000 rpm	Castor oil and soybean oil	80	70	71
	FASCAT® 4100, 4201 and 4350	MeOH : oil : catalyst = 400 : 100 : 1 with reflux at constant stirring	Soybean oil	80	43	72
	BuSn(O)OH, Bu_2_SnO, and Bu_2_Sn(Lau)_2_	MeOH : soybean oil : catalyst = 400 : 100 : 1 and reflux at 1000 rpm	Soybean oil	79	98	73
	Cd, Mn, Pb, Zn carboxylic salts	MeOH : soybean oil : catalyst is 400 : 100 : 1 and reflux at *T* = 150–200 °C, time = 1 h	Soybean oil	78	96	74

### 3.3 Hydrogenation

The hydrogenation of esters is crucial to the chemical industry^76,77^ for the synthesis of biolubricants or surfactants that have broad applications in agrochemicals, pharmaceuticals, fine chemicals and consumer goods. The reduction of aldehydes, esters and ketones by employing molecular H_2_ at higher temperatures and pressures for producing ester-based lubricants is important to our daily life. The normal ester content of high oxygen is not suitable for them to be used as fine chemicals and engine oils as they are not biodegradable. Moreover, after hydrogenation of ester-based lubricants, the oxygen content decreases and the hydrogen content increases; hence, these lubricants can be potentially used as engine oils, which satisfies all types of lubrication.

The potential catalysts that influence the reduction process of carbonyl group present in ester products include Ru, Rh, Os, or other valuable metal based complexes, like Pt, Au, Pd, Ag, Ir, In.^78,79^ However, the high price and limited availability of the precious metal-based catalysts has led to the development of less expensive active metals. Transition metal (Cu, Ni, Co, Cr and Fe) based complexes^80^ have gained recent interest. To date, organometallic complexes as catalysts for hydrogenation of esters are broadly used for creating a homogeneous phase. The ester product becomes highly hydrogenated, but catalyst separation is quite difficult. The main challenges of ligand-based catalysts are the stability of the catalysts,^81^ catalytic reactivity, and limitation for overcoming electron transfer routes that can be found in transition metal complexes.^82^

**Fig. 10:**
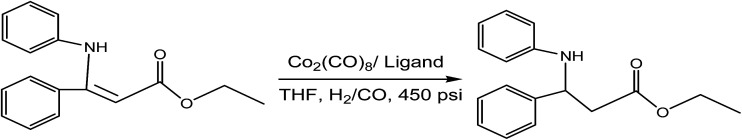
Hydrogenation of β-enamino esters under various conditions.^83^

Valencia *et al.*^83^ investigated the hydrogenation of β-enamino esters, which was catalysed by cobalt complexes with a chiral (R-BINAP) ligand, bidentate phosphine, or achiral (PPh_3_) ligand, monodentate phosphine. It was observed that the [Co_2_(CO)_8_/rac-BINAP] catalytic system exhibited a higher reactivity at 120 °C with 7–15 h of reaction time. Moreover, the catalytic efficiency of cobalt complexes in asymmetric hydrogenation reaction of β-enamines was evaluated by altering racemic ligands and is summarised in Table 20. Fig. 10 implies how one molecule of hydrogen reacted with β-enamino ester, forming a hydrogenated ester in the presence of the Co_2_(CO)_8_ catalyst and racemic ligand at 450 psi.

**Table 20:** Hydrogenation of β-enamino esters when various chiral phosphorus ligands were introduced, race = racemic ligands, Tolu = toluene, Met = methyl^83^^,^^a^

Entry no.	Metal complex	Temp. (°C)	Time (h)	Ligands	Yield (%)
01	Co_2_(CO)_8_	120	24	Race-BINAP	90
02		120	15	Race-BINAP	89
03		120	7	Race-BINAP	90
04		120	7	(*R*)-Tolu-BINAP	92
05		120	7	(*R*)-H_8_-BINAP	89
06		120	7	(*R*,*R*)-DIOP	71
07		120	7	(*R*,*R*)-Met-DuPHOS	81

a Results of conversions are not provided by the study.

Chakraborty *et al.*^84^ studied iron-based compounds as catalysts for the hydrogenation of esters to corresponding ester based-lubricants, surfactants or plasticisers bearing a PNP-pincer ligand. The PNP-pincer ligand contains a pyridine type molecule having donor atoms P, N, and P, which form complexes with iron acting as a catalyst for the hydrogenation of esters to ester-based lubricants at 115 °C under 63 psi per g of H_2_ pressure in toluene. In this study, the hydrogenation of CE-1270, derived from coconut oil, containing methyl laurate (C_12_, 73%), methyl myristate (C_14_, 26%), and a small amount of C_10_ and C_16_ methyl esters, was performed at 135 °C under 750 psig of H_2_ pressure and the iron complex was used as the catalyst (1 mol%). CE-1270 (typically 1.6–1.7 g) was fully converted in 3 h, producing ester-based oil that was confined by GC (yield; 98.6%).

**Fig. 11:**
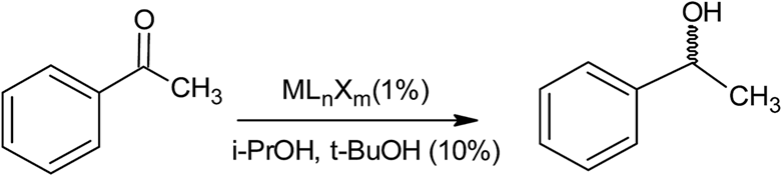
Asymmetric hydrogenation (AH) of acetophenone.^85^

Karamé *et al.*^85^ carried out an experiment on the asymmetric hydrogenation of acetophenone using N_4_-Schiff based chiral complexes containing sulfonamide or amine functionalities of ruthenium. The asymmetric hydrogenation (AH) was conducted at room temperature at 30 bar hydrogen pressure in the presence of a chiral catalyst (whereby the catalyst was synthesised by introducing the chiral ligand to the metallic precursor in absolute media). The asymmetric hydrogenation reaction of acetophenone with some metals of Rh, In, and Ru was also performed under the same conditions. The hydrogenation with the Ru species was usually carried out under solvent conditions (*e.g. tert*-butanol, iso-propanol), and the mixture was stirred with H_2_ for 24 hours (Table 21). Fig. 11 indicates that in the presence of metal complexes and i-PrOH and *t*-BuOH, the ester was converted to the corresponding hydrogenated ester (lubricant) under the mentioned reaction conditions.

**Table 21:** Asymmetric acetophenone hydrogenation with Ru, Rh, and In complexes^85^

Catalysts	Solvents	Bases	Conversion (%)	Yield (%)
[Rh(COD)_2_]OTf	MeOH	—	74	00
[Ir(COD)_2_]BF_4_	MeOH	—	56	43
[Ru(COD)Cl_2_]_*x*_	i-PrOH	*t*-BuOK	100	41
[Ir(COD)Cl]_2_	i-PrOH	*t*-BuOK	99	20
[Ru(PPh_3_)_3_Cl_2_]	i-PrOH	*t*-BuOK	100	54
[Ru(C_6_H_6_)Cl_2_]_2_	i-PrOH	*t*-BuOK	100	20

**Fig. 12:**
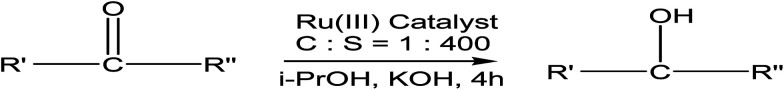
Transfer hydrogenation of ester to hydrogenated ester.^86^

Prabhu *et al.*^86^ described the transfer hydrogenation (TH) process of ketones using Ru(ii) carbonyl complexes bearing benzoylhydrazone ligands. The TH process of aromatic, aliphatic, heterocyclic, and cyclic ketones^87^ was studied by using Ru(iii) complex and iso-PrOH/KOH (base) at 82 °C and the results are summarized in Table 22. The following reaction shows the reaction scheme of the Ru(iii) complex catalysed transfer hydrogenation process of ketones to the corresponding hydrogenated ester. In this case, 4-nitro ketone and 4-cyano ketone (entry 1 and 2) were converted to their corresponding hydrogenated esters (about 99%) efficiently in the presence of Ru(iii) complexes in basic media (i-PrOH/KOH) (Fig. 12).

**Table 22:** Transfer hydrogenation (TH) of ketones using Ru(iii) complex^86^^,^^a^

Entry no.	Ketones	Hydrogenated ester	Conversion (%)	TON	TOF
1	4-Nitro ketone	4-Nitro ester	99.50	796	199
2	4-Cyano ketone	4-Cyano ester	99.00	792	198
3	4-Bromo ketone	4-Bromo ester	98.4	787	197
4	Acetophenone	Benzyl ester	97.5	780	195
5	4-Methyl ketone	4-Methyl ester	96.3	770	193
6	4-Hydroxy ketone	4-Hydroxy ester	92.7	742	185

a TOF = turn over frequency, TON = turn over number.

**Fig. 13:**
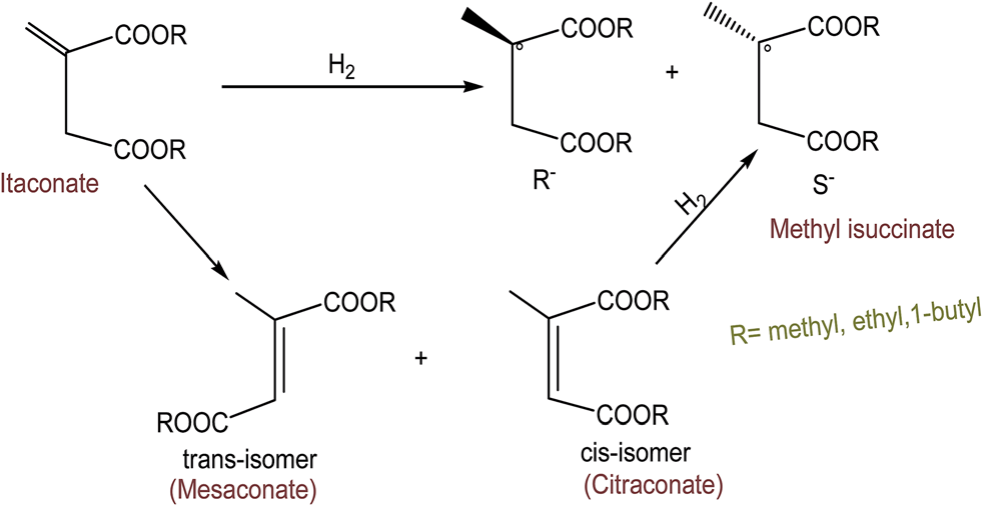
Asymmetric hydrogenation of itaconates.^88^

Shimazu *et al.*^88^ esterified esters of α,β-unsaturated fatty acids through asymmetric hydrogenation using supported Rh(i)–phosphine on smectites and supported Rh hectorite (H) complex in acetonitrile/H_2_O solution. The study found that the asymmetric selectivity depends on the solvents, bulkiness of the ester groups and the interaction of substrate molecules. Hectorites could be incorporated into metal complexes as well as many organic compounds. Since tempered clays have certain interlayer sites, the interaction of chiral ligands with the substrates could occur. Such potential interaction may increase the selectivity in the asymmetric reactions. The proposed mechanism for hydrogenation is shown in Fig. 13. Itaconate has two forms: *trans* isomer of itaconate is known as mesaconate and *cis*-isomer is known as citraconate. *cis*-Isomer (*i.e.*, citraconate on hydrogenation) gives the corresponding saturated hydrogenated ester. The high selectivity was found for the hectorite catalyst in all the solvents compared to that for the homogeneous catalysts, since hectorite still maintained its layer structure even after being swollen with the solvents. The most recommended operating parameters for the mentioned hydrogenation process in the suggested catalyst were hydrogen pressure (*P*_H_2__) = 1.01 × 10^5^ Pa; temperature (*T*) = 30 °C; substrate concentration = 6.25 × 10^−4^ mol; and substrate/Rh = 100 (Table 23).

**Table 23:** Hydrogenation of diesters of itaconate & dibutyl mesaconate using [Rh((S)BINAP)(COD)]^+^/H at different solvents^88^^,^^a^

Catalysts	Substrates	Solvents	Time (h)	Conversion (%)
[Rh((S_1_)-BINAP)L_2_]^+^/H	Methyl	MeOH	2	100
	1-Butyl	MeOH	24	98
[Rh((S_1_)-BINAP)L_2_]CIO_4_	Methyl	MeOH	2	100
	1-Butyl	MeOH	12	93
[Rh((S_1_)-BINAP)L_2_]^+^/H	1-Butyl (Mesa)	Et/Cy	312	100
[Rh((S_1_)-BINAP)L_2_]CIO_4_	1-Butyl (Mesa)	Et/Cy	74	100
[Rh((S_1_)-BINAP)L_2_]^+^/H	Methyl (Citra)	Et/Cy	46	96
[Rh((S_1_)-BINAP)L_2_]CIO_4_	Methyl (Citra)	Et/Cy	2.5	100

a Reaction parameter: *P*_H_2__ = 1.01 × 10^5^ Pa, *T* = 30 °C, L_2_ = cyclooctadiene Et = ethanol, Cy = cyclohexane, Et/Cy = 2, Mesa = mesaconate, Citra = citraconate.

Table 24 summarises different catalysts used for the hydrogenation process in order to produce hydrogenated esters. The potential catalysts for this purpose are valuable metals like Ru, Rh, Os, Pt, Au, Pd, and Ir based complexes. In fact, the high price and limited availability of these metal-based catalysts leads to the development of highly abundant and less expensive active metals, *i.e.*, transition metal (*e.g.*, Cu, Ni, Co, Mn and Fe) based complexes are gaining recent interest. Nowadays, transition metal complexes as catalysts for hydrogenation of esters are widely used for producing ester-based lubricants.

**Table 24:** Catalysts for hydrogenation reaction

Catalyst type	Specific catalyst	Reaction conditions	Feedstock	Conv. (%)	Ref.
Homogeneous	Co_2_(CO)_8_	Enamine = 0.37 mmol, ligand = 0.0075 mmol, THF = 10 mL, ratio of H_2_/CO (1 : 3, 450 psi) at 120 °C	β-Enamino ester	90	83
	Iron based pincer complexes	*T* = 135 °C, *P*_H_2__ = 750 psi per g, catalyst = 1 mol, CE-1270 = 1.6–1.7 g, time = 3 h	Methyl laurate and methyl myristate	95	84
	Ru(PPh_3_)_3_Cl_2_	[S] = 0.3 M & S : k-BuOK : L : M = 100 : 10 : 1 : 1; *P*_H_2__ = 30 bar *T* = 50 °C for 16 h	Acetophenone	100	85
	Ru(L)(CO)(EPh_3_)_2_	Ketone = 2.4 mmol, complex 2 = 3 μmol, KOH = 12 μmol and *m*-xylene = 0.24 mmol in i-PrOH, *T* = 82 °C and reflux time = 4 h	Acetophenone	94	86
Heterogeneous	[Rh((S_1_)-BINAP)(COD)]^+^/H & [Rh((S_1_)-(R)-BPPFA)(COD)]^+^/h	Solvent = 3 mL; *P*_H_2__ = 1.01 × 10^5^ Pa; and *T* = 30 °C; substrate = 6.25 × 10^−4^ mol; subs/Rh = 100; L_2_ = COD	Carboxylic acid esters	95	88

### 3.4 Hydrogenation–esterification reaction

In the one-step hydrogenation–esterification (OHE) process, biooils are converted into biofuels (biolubricants) simultaneously in the same reaction vessels employing the same catalyst and reaction conditions. The final products become highly suitable for combustible hydrocarbons as they are hydrogenated to form esters *via* basic reactions such as esterification^89,90^ and hydrogenation.^91,92^ The main constituents of biooils normally employed for the one-step hydrogenation–esterification process are fatty acids, aldehydes and ketones, and phenols, which affect the properties of biooils negatively. For instance, acetic acid, levulinic acid, furfural, hydroxyacetone, phenol, and ethanediol are considered as the recommended raw materials^93^ for production of biolubricants. The OHE process utilises different bifunctional metals-based catalysts,^94^ such as RANEY® Ni (RN) or Lewis acid catalyst under solvent conditions (methanol/ethanol) in order to generate combustible and stable compounds, *i.e.* hydrogenated esters.

Xu *et al.*^95^ investigated a method to convert biooils into the hydrogenated ester using RN catalyst for the hydrogenation–esterification reaction. Acetic acid, furfural and hydroxyacetone were used as model compounds and 100% conversion of hydroxyacetone and furfural were attained over Ni catalysts modified with Mo, Sn, Fe, and Cu in the presence of methanol. The rate of conversion of CH_3_COOH was 35.1% when methanol was not added, but when 6 g/8 g methanol/biooil was added, the rate of conversion of acetic acid increased to 81.1%. Table 25 indicates that when Mo–RN was modified by 5% Fe, the conversion of methanol decreased from 35.6% to 28.6%. The degree of decrease was not as significant as the one of Fe–RN. However, the conversion of phenol decreased significantly by addition of Fe as compared to Mo–RN.

**Table 25:** Conversion of bio-oils over modified Mo–Ni with different Fe contents^95^^,^^a^

Model compounds	Conversion (%)		
	Fe–Mo–RN (0%)	Fe–Mo–RN (1%)	Fe–Mo–RN (5%)
Methanol	35.60	37.10	28.60
Acetic acid	81.30	83.90	82.70
Phenol	76.90	49.90	51.90
Furfural	100	100	100
Ethanediol	7.00	20.70	9.50
Hydroxy acetone	100	100	100

a Reaction conditions: 0.5 g of Mo–Ni, temp. = 180 °C, H_2_ pressure = 5 MPa, and batch reaction time = 4 h.

Yu *et al.*^96^ described the bio-oil upgrading by the hydrogenation–esterification process using acetic acid and furfural as model compounds over bifunctional Pd. The OHE was performed in a 100 mL stainless steel autoclave with an equimolar ratio of furfural and acetic acid, with 0.40 g catalyst (0.40 Pd/C + 0.40 g Al_2_(SiO_3_)_3_ for mixed bifunctional catalyst) by adding toluene solvent. Reaction time of 4 h at 1.0–4.0 MPa of H_2_ and 80–200 °C with a stirring speed of 800 rpm and a particle size of 400 meshes were adopted to evaluate the catalytic performances of the tested catalysts. The catalytic performances of the catalysts used for OHE reaction of FAL and HAc is listed; among the tested catalysts, 5% (v/w) Pd/Al_2_(SiO_3_)_3_ showed the best catalytic activity. The better OHE activities over the composite bifunctional catalysts may be attributed to their better cooperative effects among metal sites and acid sites.

Yu *et al.*^97^ studied Al-SBA-15 supported Pd bifunctional catalysts (Pd/C, Pd, *etc.*) to upgrade bio-oils using acetic acid and furfural as the model compounds *via* the OHE process. The OHE experiments were conducted with an equimolar mixture of 0.10 mol furfural and 0.10 mol acetic acid, which were dissolved in 10.0 mL toluene and 0.40 g of Pd/Al-SBA-15 (5%) or 0.40 g Pd/C (5%) + 0.40 g Al-SBA-15 catalyst was added to the reactor. The reaction was performed under optimum condition of 2.0 MPa H_2_ pressure, 150 °C of reaction temperature, stirring speed of 800 rpm for 4 h of reaction time. It was important that the synergistic effect between the metal sites and the acid sites on bifunctional Pd/Al-SBA-15 (5%) favoured the OHE reaction, and the following results were obtained. Table 26 summarises the effects of the acidity on the support materials, as the reaction activity was studied over 5% Pd/Al-SBA-15(*X*) with different Si/Al ratios and the catalytic results are also shown. It is illustrated that with increasing Si/Al of Al-SBA-15 (decrease in acidity of the supports), *Z*_(FAL)_ (conversion of furfural) decreases but *X*_(D)_ (yield) increases.

**Table 26:** Catalytic activities for OHE reaction of furfural and acetic acid^97^^,^^a^

Catalysts (5%)	*Z*_(FAL)_ (%)	*Y*_(FOL)_ (%)	*Y*_(FA)_ (%)	*Y*_(BP)_ (%)	*X*_(D)_ (%)
Pd/C + Al-SBA-15(300)	70.70	43.10	16.00	40.90	41.80
Pd/Al-SBA-15(100)	71.90	56.90	16.50	26.60	52.80
Pd/Al-SBA-15(22)	73.20	49.30	15.80	34.90	47.70
Pd/Al-SBA-15(300)	70.30	61.80	18.20	20.00	56.20
Pd/SBA-15	35.20	92.20	3.30	4.50	33.60
Pd/Al-SBA-15(500)	48.60	79.60	9.60	10.80	43.40
Pd/C + Al_2_(SiO_3_)_3_	69.40	19.70	9.10	71.20	20.0

a *Z*_(FAL)_ – conversion of furfural, *Y*_(FOL)_ – selectivity of furfuryl alcohol, *Y*_(FA)_ – selectivity of furfuryl acetate, *Y*_(BP)_ – selectivity of by-products, *X*_(D)_ – yield to desired products.

Tang *et al.*^98^ investigated the catalytic activity of bifunctional Pd and Pt catalyst loaded with acidic supports by upgrading the bio-oils through the OHE route using fatty acid and aldehyde as the starting material. Acetic acid and butyl aldehyde were selected as model compounds for the OHE process using Pt catalysts with supported HZSM-5 and/or amorphous aluminium silicate as the bifunctional catalysts, which means they exhibited the properties of hydrogenation and esterification. The catalysts with a large surface area, high pore volume, small particle size, and strong acidic nature may be favourable for OHE reaction. The reaction was performed at 150 °C with hydrogen pressure of 15 atm, 0.2 g of catalyst, and reaction time of 4 h with a stirring speed of 750 rpm.

From the above study, the most recommended catalyst for the hydrogenation–esterification process of bio-oil upgrading are Pt catalysts with acidic supports *e.g.*, HZSM-5 or amorphous aluminium silicate. These catalysts are bifunctional, have a large surface area, high pore volume, small particle size, and strong acidic nature and are the most favourable for the one-step hydrogenation–esterification (OHE) reaction. The better OHE activities of the bifunctional catalysts may be attributed to its better cooperative effect with metal sites and acid sites. The OHE reaction between aldehyde and acid to ester is feasible over a bifunctional catalyst under high temperature and pressure for producing biolubricants (Table 27).

**Table 27:** Catalysts for hydrogenation–esterification reaction

Catalyst type	Specific catalyst	Reaction conditions	Feedstock	Conv. (%)	Ref.
RANEY® Ni	RANEY® Ni with Mo, Sn, Fe, Cu	Mo–RN = 0.5 g, *T* = 180 °C, *P*_H_2__ = 5 MPa, reaction time = 4 h	Acetic acid and furfural	80	95
Bifunctional Pd	Pd/Al_2_(SiO_3_)_3_ (5%) and Pd/C + Al_2_(SiO_3_)_3_ (5%)	*T* = 150 °C, *P*_H_2__ = 2.0 MPa, rpm = 800, time = 4 h, 9.60 g FAL + 6.00 g HAc + 0.40 g (or 0.40 g Pd/C + 0.4 g Al_2_(SiO_3_)_3_ in entry 1) in 10.0 mL Tolu	Furfural and acetic acid	66.4	96
Bifunctional Pd/Al-SBA-15	5% Pd/Al-SBA-15 and 5% Pd/Al_2_(SiO_3_)_3_	*T* = 150 °C, *P*_H_2__ = 2.0 MPa, rpm = 800, time = 4 h, 9.60 g FAL + 6.00 g HAc + 10.0 mL toluene	Furfural and acetic acid	70	97
Pt with acidic supports	5% Pt/HZSM-5 and 5% Pt/Al_2_(SiO_3_)_3_	*T* = 150 °C, catalyst = 0.2 g, butyl aldehyde = 18 g, acetic acid = 15 g, time = 4 h, stirring speed = 750 rpm	Acetaldehyde and acetic acid	91	98

## 4 Future prospects and conclusion

The increasing demand towards renewable and sustainable energy has *attracted the interest of researchers to develop sustainable biolubricants for automotive and machinery applications. Studies revealed that biolubricants exhibit a better price-performance ratio compared to conventional mineral oils, as they can adhere on metal surfaces for a longer time. This review demonstrates that biolubricants are potentially utilised as alternative lubricants for automobile applications such as engine oils, hydraulic and metal working fluids, and grease and chainsaw oils. Our review indicates that metal complex catalysts are potentially used to catalytically synthesise biolubricants as high Lewis catalytic activity of metal complex catalysts were observed in the reactions. For esterification and transesterification, tin complexes of Sn(ii) and Sn(iv) catalysts were found to have enhanced the reaction significantly, as they exhibited Lewis acid character and the vacant 5d orbital was well coordinated with subtract molecules in the reaction mixture. Meanwhile, the bifunctional catalyst of 5% (w/w) Pt/Al_2_(SiO_3_)_3_ had the highest activity for ester formation *via in situ* hydrogenation–esterification reaction due to its large surface area, high pore volume and strong acidic nature, supplemented with 91% yield. In addition, research findings showed that the transition metal catalysts could maximise the selectivity of biolubricants and minimise the formation of undesirable products.

To date, obtaining suitable and well-performed lubricants for specific engines operation is challenging in the present lubricant industry. Therefore, it is essential to develop new-generation lubricants for industries to meet the demand of lubricated automotives. Biodegradable and non-toxic lubricants can reduce environmental pollution by lowering carbon emission. In addition, the good lubricating properties, high load-carrying capacity, longer service life, and rapid biodegradability of biolubricants have enhanced the current field of interest.

Furthermore, the development of new lubricant additives, which give a better lubrication under extreme conditions, is another important challenge in the biolubricant field. The new transition metal complexes act as a homogeneous catalyst for upgrading biomass and non-edible oil based lubricants for automotive applications. Nevertheless, considerable works is yet to be accomplished in this field, especially in the development of a new and economic method to develop transition metal-complex catalysts. Moreover, the application of potential metal complex catalysts by upgrading biomass in ester-based lubricants is necessary. The development of effective catalysts for various types of biolubricants are vital and have been recognised to perform better than conventional petroleum oils in modern automotive applications.

## Conflicts of interest

There are no conflicts to declare.

## Supplementary Material
